# Fluorescein-Guided Panendoscopy for Head and Neck Cancer Using Handheld Probe-Based Confocal Laser Endomicroscopy: A Pilot Study

**DOI:** 10.3389/fonc.2021.671880

**Published:** 2021-06-14

**Authors:** Andreas Dittberner, Rafat Ziadat, Franziska Hoffmann, David Pertzborn, Nikolaus Gassler, Orlando Guntinas-Lichius

**Affiliations:** ^1^ Department of Otorhinolaryngology, Jena University Hospital, Jena, Germany; ^2^ Section of Pathology, Institute of Forensic Medicine, Jena University Hospital, Jena, Germany

**Keywords:** head and neck surgery, confocal endomicroscopy, laser, fluorescein sodium, fluorescence guided surgery, optical biopsy

## Abstract

**Background:**

White-light endoscopy and microscopy combined with histological analysis is currently the mainstay for intraprocedural tissue diagnosis during panendoscopy for head and neck cancer. However, taking biopsies leads to selection bias, *ex vivo* histopathology is time-consuming, and the advantages of *in-vivo* intraoperative decision making cannot be used. Confocal laser endomicroscopy (CLE) has the potential for a rapid and histological assessment in the head and neck operating room.

**Methods:**

Between July 2019 and January 2020, 13 patients (69% male, median age: 61 years) with newly diagnosed head and neck cancer (T3/T4: 46%) underwent fluorescein-guided panendoscopy. CLE was performed from both the tumor and margins followed by biopsies from the CLE spots. The biopsies were processed for histopathology. The CLE images were *ex vivo* classified blinded with a CLE cancer score (DOC score). The classification was compared to the histopathological results.

**Results:**

Median additional time for CLE during surgery was 9 min. A total of 2,565 CLE images were taken (median CLE images: 178 per patient; 68 per biopsy; evaluable 87.5%). The concordance between histopathology and CLE images varied between the patients from 82.5 to 98.6%. The sensitivity, specificity, and accuracy to detect cancer using the classified CLE images was 87.5, 80.0, and 84.6%, respectively. The positive and negative predictive values were 87.0 and 80.0%, respectively.

**Conclusion:**

CLE with a rigid handheld probe is easy and intuitive to handle during panendoscopy. As next step, the high accuracy of *ex vivo* CLE image classification for tumor tissue suggests the validation of CLE *in vivo*. This will evolve CLE as a complementary tool for *in vivo* intraoperative diagnosis during panendoscopy.

## Introduction

Panendoscopy as part of the staging of a patient with suspected head and neck cancer involves examination of the nasopharynx, oral cavity, oropharynx, hypopharynx, larynx, esophagus, and lung, performed under general anesthesia. It aims to confirm the malignant diagnosis, determine the extent, accessibility, resectability of the primary tumor and synchronous primary tumors (1). The mucosa is evaluated with white light, using direct visual inspection, endoscopy, and microscopy. Based on this experience, the head and neck surgeon takes biopsies from the suspected tumor area. Biopsies of the tumor border area are important to determine the tumor extent and resectability. Hence, the information received by use of frozen section during the surgery or by final histopathology is limited to areas where biopsies are taken and can lead to a selection bias. Furthermore, the accuracy of frozen sections in the head and neck region is limited (2). Basically, the same holds true for tumor border definition during definitive tumor surgery. A complete tumor resection with clear margins (R0) is an important prognostic factor in head and neck oncology (3). Currently, the described standard setting leads to a R1 resection rate in 7.5–10% of cases, of which 50% subsequently require reoperation and/or radiotherapy. The other 50% are undetected R1 resections, of which approximately 75% develop a relapse within 2 years (4). A better intraoperative determination of tumor boundaries during panendoscopy and definitive ablative tumor surgery is urgently needed. Hence, the development of new innovative techniques to improve the determination of tumor boundaries is therefore of great interest (5).

One of these innovative technologies is confocal laser endomicroscopy (CLE). Using fluorescein as a contrast agent, CLE visualizes the cellular microstructure of the superficial mucosal layer with a high resolution. Fluorescein distributes within intercellular spaces and outlines the mucosal cells enabling a structural analysis of the cellular texture (6). Because CLE was so far most frequently used in the field of gastroenterology, most CLE systems use flexible probes to be applied *via* the working channel of a flexible gastroscope. This flexible system was also used for analysis of head and neck cancer (7–12). Recently, a rigid probe base system was introduced primary applied in neurosurgery (13–15). The newest generation of this rigid probe-based CLE system has a Z-stack function resulting in a series of images allowing a three-dimensional reconstruction. Without changes of the position, a series of CLE images is acquired starting from the surface and ending at the deepest position, which might allow a better visualization and analysis of the tissue (16–18).

The rigid handheld construction of the CLE probe also is of primary interest for application in head and neck surgery. In a study in 2016 at our department, a flexible probe-based CLE system without a handheld function was used. At this time, we had to fixate the flexible probe in a rigid metal suction tube for an optimal contact to the superficial mucosal layer during panendoscopy, which was essential for recording CLE images of minimum standard quality. Even better experienced after the first panendoscopies, a standard CLE image quality was still hard to display with the flexible probe at adverse angled spots in the upper airways (11). The handheld rigid probe of the newest CLE system seems to be better and intuitively applicable during our first applications in diagnostic panendoscopies. An increased number of CLE images with a high standard quality seems to be created in a shorter time compared to the flexible probe-based CLE system. One important reason is the design of the tip of the probe. The probe makes it far easier to get an optimal contact to the superficial mucosal layer. Herein, we describe how we combined the handheld probe with a simply usable retention arm system to place the tip of the probe at a single spot of the superficial mucosal layer and without furthermore moving of the probe. At each spot, we could additionally apply the new Z-stack function of the CLE system, which created an image stack usable for a 3D reconstruction of the superficial mucosal layer. In this pilot study, we share a practicable workflow for the routine use of a rigid handheld probe-based CLE application during panendoscopy and how to create high quality image stacks for a 3D reconstruction of the superficial mucosal layer. We also want to report our first findings of the accuracy to detect head and neck cancer.

## Methods

This prospective study established a workflow for the intraoperative use of CLE and evaluated our clinical experience using CLE during head and neck cancer surgery between July 2019 and January 2020 at the Department of Otorhinolaryngology, Head and Neck Surgery, Jena University Hospital, Jena, Germany. The study was approved by the local ethics committee of the Jena University Hospital (No. 3008-12/10). Patients scheduled for panendoscopy because of head and neck cancer were prospectively enrolled in this study. Patient inclusion criteria were as follows: Age ≥18 years, primary untreated head and neck squamous cell carcinoma, and written informed consent. Exclusion criteria were as follows: pregnancy, previous documented adverse reaction or allergy to fluorescein, severe renal impairment (glomerular filtration rate <44 ml/min/1.73 m^2^), or hepatic impairment (Child-Pugh ≥B).

### Imaging Protocol

**Figure 1:**
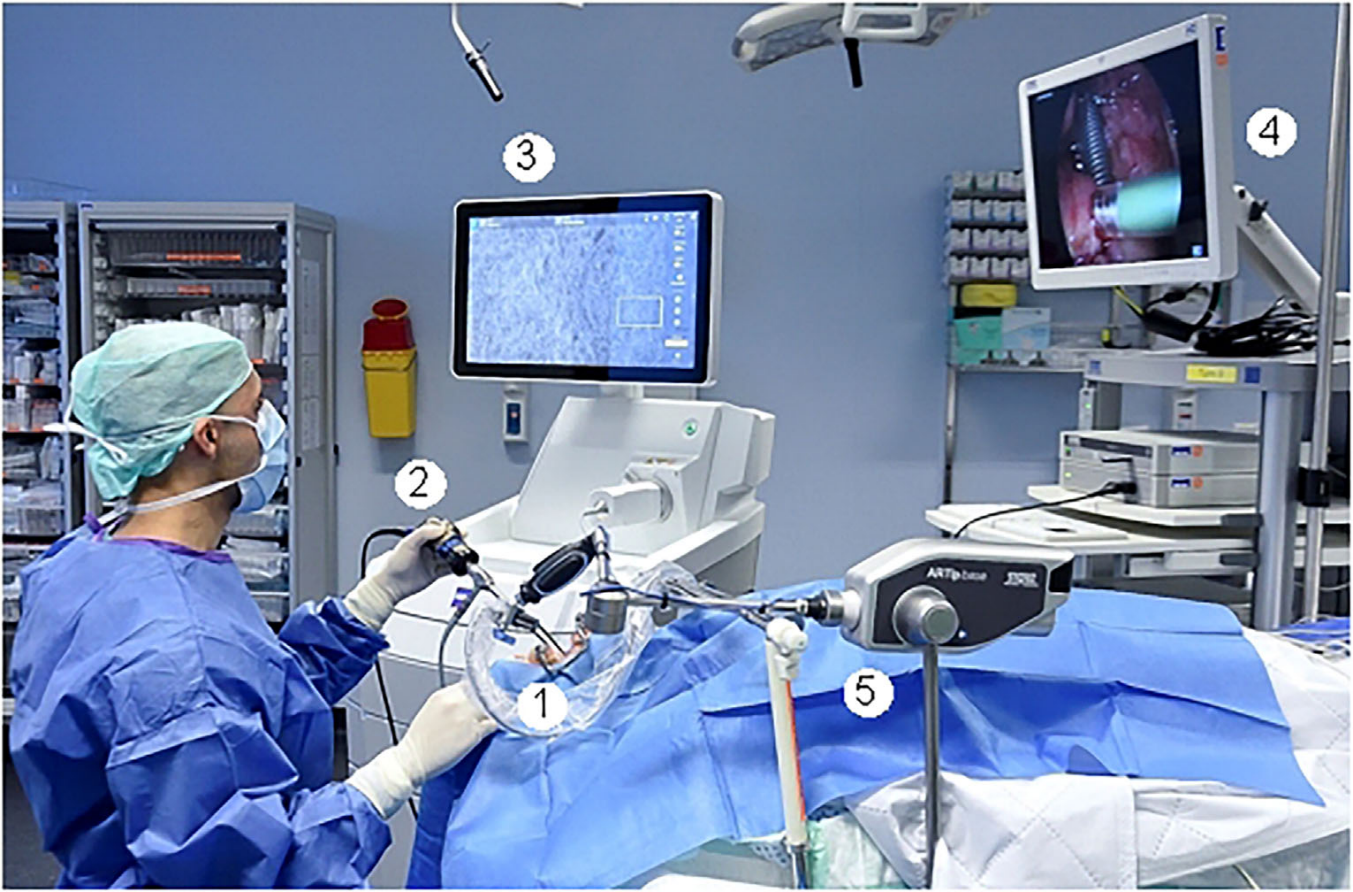
Intraoperative setting. 1: Fixed CLE probe. 2: White light endoscope with attached camera. 3: Screen with the CLE image. 4. Screen with the endoscopic white light image. 5: Holding arm for fixation of the CLE probe.

**Figure 2:**
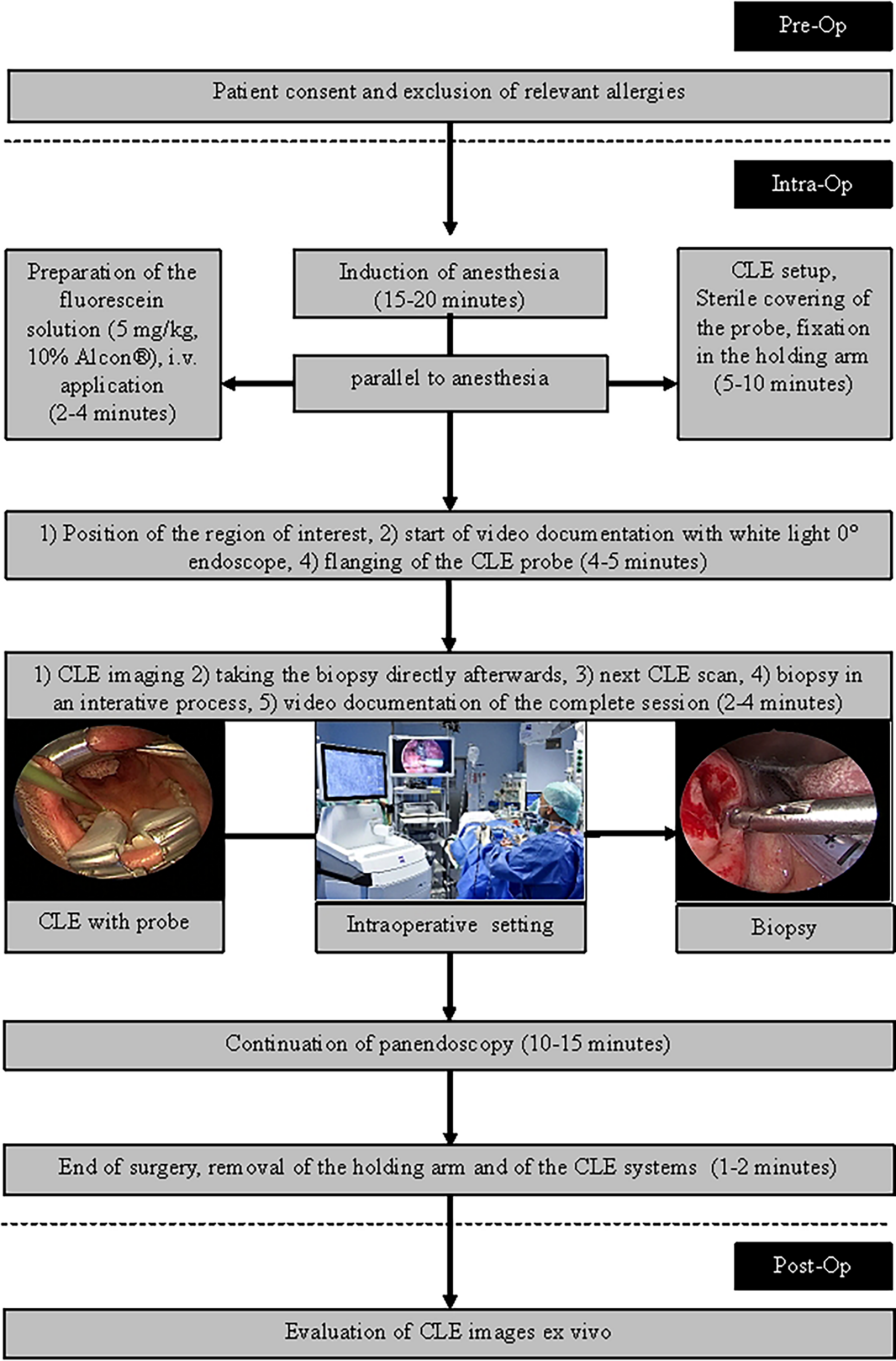
Workflow for the CLE imaging during panendoscopy.

The intraoperative setting is shown in **Figure 1** and in **Figure 2**. A handheld probe-based portable clinical-grade CLE system (CONVIVO, Carl Zeiss AG, Oberkochen, Germany) was used. The miniaturized scanner consisted of a handheld and rigid probe with an outer diameter of 5 mm and a working length of 150 mm. The scan field had a dimension of 475 µm (horizontal) and 267 µm (vertical) with an image resolution of either 1,920 × 1,080 pixels (full high definition [HD]), 0.75 images per second) or 1,920 × 270 pixels (2.35 images per second). The laser endomicroscope had a scanning depth of 250 µm. An automatic Z-stack acquisition function allowed to stack two-dimensional 35 full HD CLE images. The distance between the stacked CLE images was 3 µm. The 3D reconstructions of a z stack were performed using an open source image processing software (ImageJ, Version 2.0.0-rc-69/1.52p, https://imagej.nih.gov/ij/index.html). The probe was covered with a sterile sheath (Carl Zeiss AG, Oberkochen, Germany).

To minimize movement artifacts during the scanning, the probe was fixed with an electric retention arm system (Artip Base, serial number 28272, Karl Storz, Tuttlingen, Germany). A rigid zero-degree endoscope (Hopkins optics 0°, 5.8 mm diameter, length 19 cm, serial number 8710AGA, Karl Storz, Tuttlingen, Germany) was used for standard white light display. After induction of anesthesia as part of regular panendoscopy, 5 mg/kg body weight 10% fluorescein (Alcon Pharma, Freiburg 10%, 100 mg/ml) was administered intravenously. First, the regular panendoscopy was carried out. The CLE examination started about 10–20 min after the fluorescein injection. First, the tip of the CLE probe was placed on the region of interest; i.e. the center of the tumor and/or in the surrounding tumor-free suspected margins. The position of the tip of the confocal probe was adjusted based on the quality of the live images on the monitor until a sufficient quality was achieved and because of using the retention arm there was no furthermore change of position during the image recording. After the CLE recording at one spot was performed, a subsequent tissue sampling by taking a biopsy at each recorded CLE spot was done. For evaluation purposes, the positioning of the CLE probe at each spot and the procedure of taking the tissue biopsy directly after the CLE recording at the same spot was extra video documented (IMAGE1 S, Karl Storz, Tuttlingen, Germany). Single CLE images and image stacks at the same spot were taking systematically from suspected tumor areas and the surrounding mucosa.

### Matching of Histopathological and CLE Images, CLE Image Classification

For histopathological analysis, all biopsies were prepared following routine protocols. The tissues were fixed in formalin at least for 12 h and the transferred into paraffin to get orthogonal slides. From the FFPE tissue, slides of about 3 µm were cut using routine microtomes and stained with hematoxylin and eosin (H&E). Representative images were done from each slide to illustrate the main histopathological information. Using the histopathology reports of all biopsies, each CLE image was annotated as a) carcinoma; b) normal tumor-free tissue, and c) chronic inflammation. Not knowing the histopathology results, two examiners (AD; RZ) classified each CLE image according to the DOC-Score (12; **Supplementary Table 1**). This score evaluates criteria of the tissue architecture, cell morphology, fluorescence leakage, and the vessels. Additionally, each image was evaluated in regard of artifacts (no/minimal artifacts, movement artifacts, blood/saliva contamination).

### Statistical Analysis

The statistical analysis was performed with SPSS version 25.0 (IBM, Armonk, NY, USA). If not indicated otherwise, data are presented with mean values ± standard deviation (SD). Histology results were set as gold standard. Sensitivity, specificity, diagnostic accuracy, and negative and positive predictive values [with 95% confidence intervals (95% CI)] to diagnose cancer with CLE scoring were evaluated.

## Results

### Patients’ Characteristics

A total of 13 patients were included (69.3% male; median age: 61 years). More details are shown in **Tables 1**, **2**. The majority of suspected lesions were located in the oropharynx (52.9%), followed by the oral cavity (35.3), and the hypopharynx (11.8%).

**Table 1:** Patients’ and histopathological characteristics.

Parameter	Absolute (N)	Relative (%)
All	13	100
Gender		
Male	9	69.3
Female	4	30.7
Localization		
Oral cavity	6	35.3
Oropharynx	5	52.9
Hypopharynx	2	11.8
T classification		
T1	4	30.8
T2	3	23.1
T3	2	15.4
T4	4	30.8
	**Mean ± SD**	**Median, range**
Age, years,	61.9 ± 7.4	61, 50–74
Duration of CLE, min	9 ± 6.9	9, 1.2–40
Biopsies per patient	2.38 ± 2.13	2, 1–9

**Table 2:** TNM staging of the 13 patients with head and neck cancer.

No.	Localization	TNM	Biopsies	Histopathology			CLE images			
				Carcinoma	Inflammation	Normal	All		Fitting* images	
			**N**	**N**	**N**	**N**	**N**	**%**	**N**	**%**
1	Oral cavity	pT1pN2M0	1	0	1	0	178	5.3	147	82.5
2	Oral cavity	pT1pN0M0	4	3	0	1	594	17.8	574	96.6
3	Oral cavity	pT1pN0M0	2	1	1	0	255	7.6	227	89.0
4	Oropharynx	pT1pN0M0	2	0	2	0	238	7.2	197	83.1
5	Oropharynx	pT2pN1M0	3	2	0	1	364	10.9	348	95.8
6	Oral cavity	pT2pN0M0	9	4	1	4	878	26.3	773	88.0
7	Oropharynx	pT2pN1M0	1	0	0	1	45	1.4	41	91.1
8	Oral cavity	pT3pN0M0	2	0	0	2	186	5.6	169	90.8
9	Oral cavity	cT3cN3M0	1	1	0	0	109	3.3	107	98.1
10	Oropharynx	cT4cN1M0	2	1	0	1	297	8.8	279	94.0
11	Oropharynx	pT4pN0M0	1	1	0	0	73	2.1	72	98.6
12	Hypopharynx	pT4pN1M0	1	0	1	0	53	1.6	45	84.0
13	Hypopharynx	cT4bN0M0	1	1	0	0	68	2.1	67	98.5
**Sum**			30	14	6	10	3,338	100	3,046	91.3
**Mean ± SD**			2.3 ± 2.2	1.1 ± 1.3	0.5 ± 0.7	0.8 ± 1.2	256 ± 242	7.7 ± 7.2	234 ± 210	91.5 ± 5.9

*CLE images with characteristic CLE parameters fitting to the histopathology.

### CLE Imaging and Correlation to the Histopathology Results

**Figure 3:**
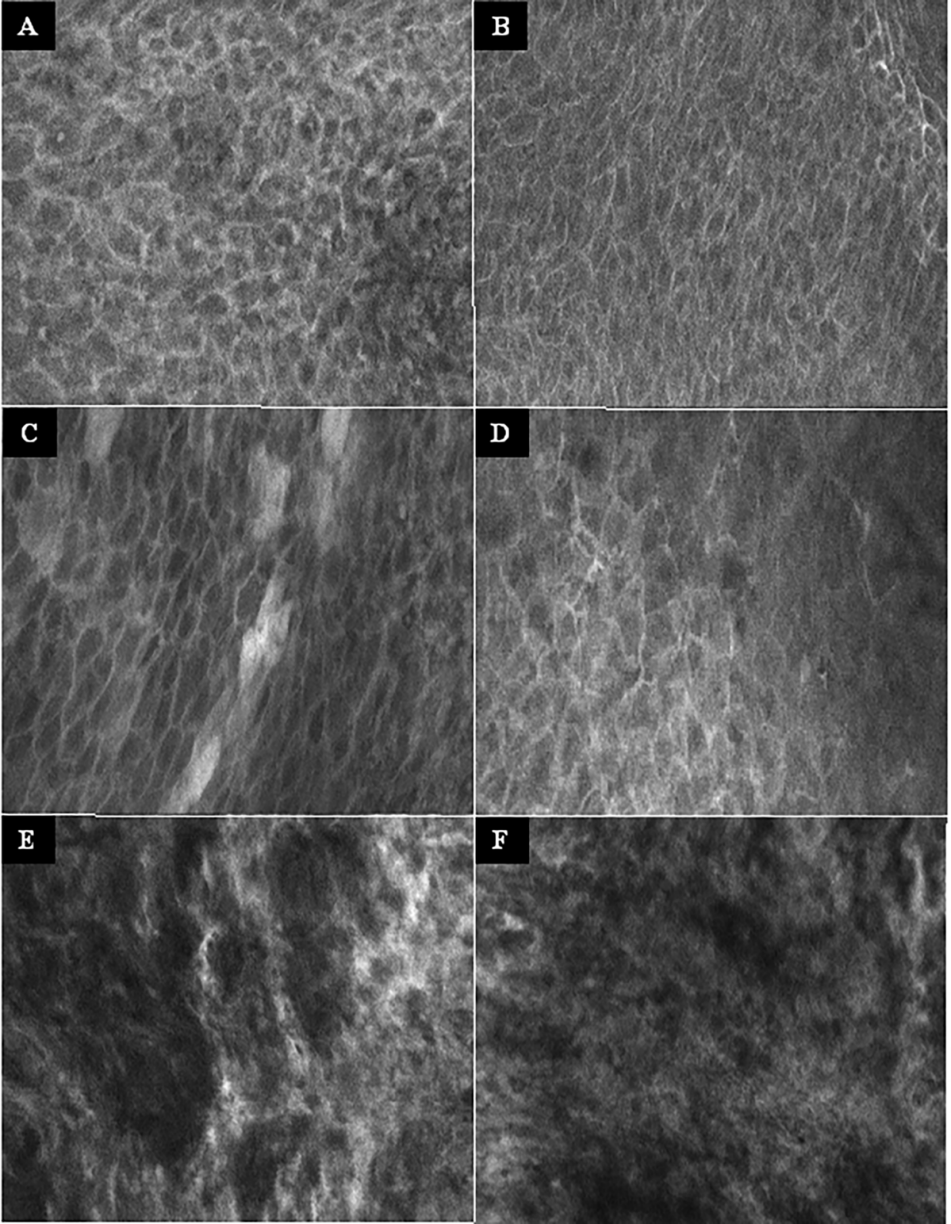
Examples of the CLE imaging. **(A–B)** Normal mucosa of the oropharynx. Intact and regular intercellular gaps, homogenous cellular architecture, intact cell walls, no fluorescein leakage. **(C–D)** Chronic inflammation of the mucosa in the oropharynx. CLE characteristics not different to normal mucosa. **(E–F)** Squamous cell carcinoma of the oropharynx. Completely unorganized tissue architecture, intercellular gaps non-existent, cell morphology irregular, black spots, fluorescein leakage. CLE image size: 464 × 261 µm, 1,920 × 1,080 pixel.

**Figure 4:**
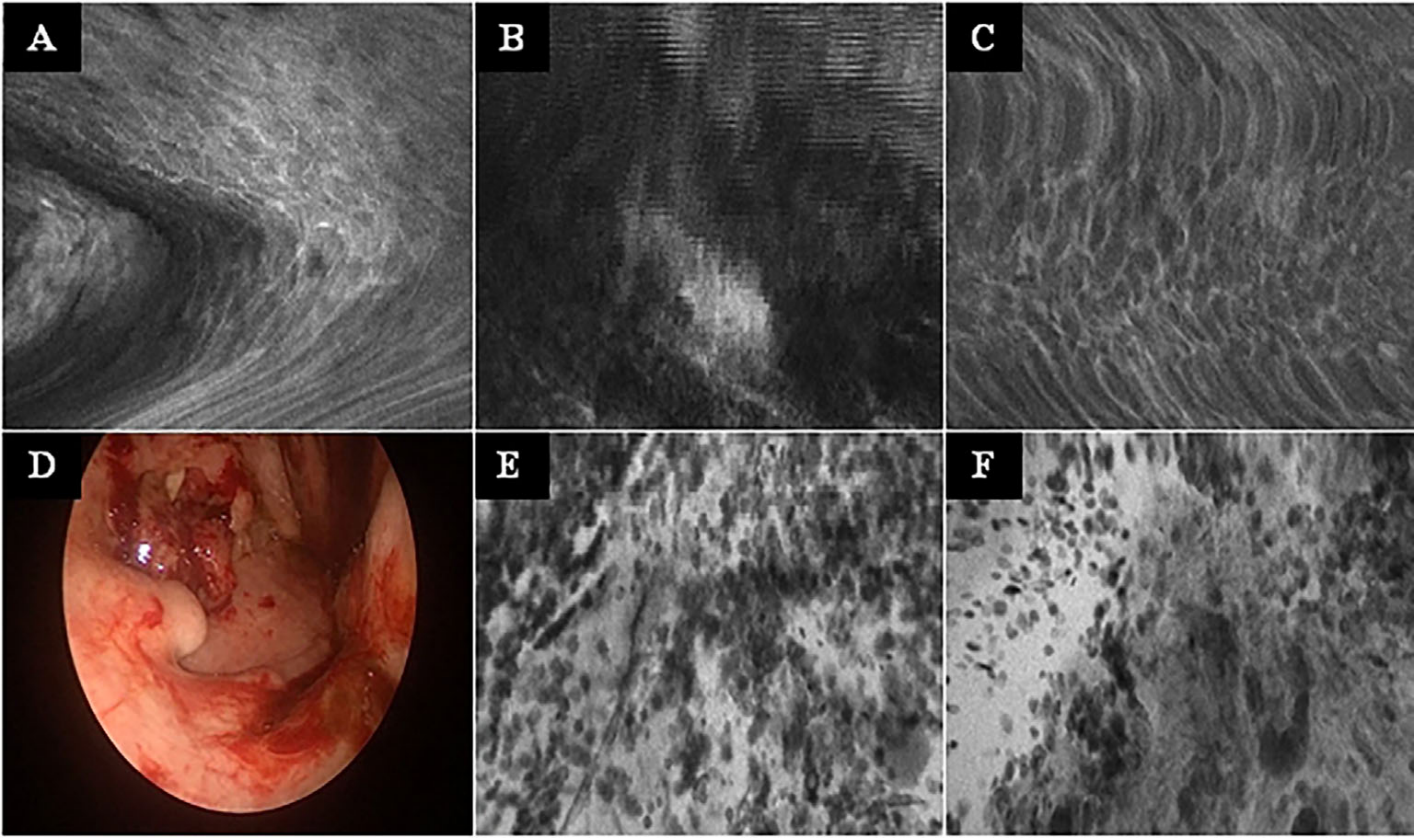
Typical CLE artifacts. **(A–C):** Movement artifacts of CLE images of normal mucosa in the oropharynx **(A)** and oral cavity **(C)**, and of a carcinoma of the oropharynx **(B)**. **(D)** Tonsil cancer with blood contamination. **(E, F)**: same tumor as in F showing artifacts due to blood and saliva contamination (whitish areas). CLE image size: 464 × 261 µm, 1,920 × 1,080 pixel.

**Figure 5:**
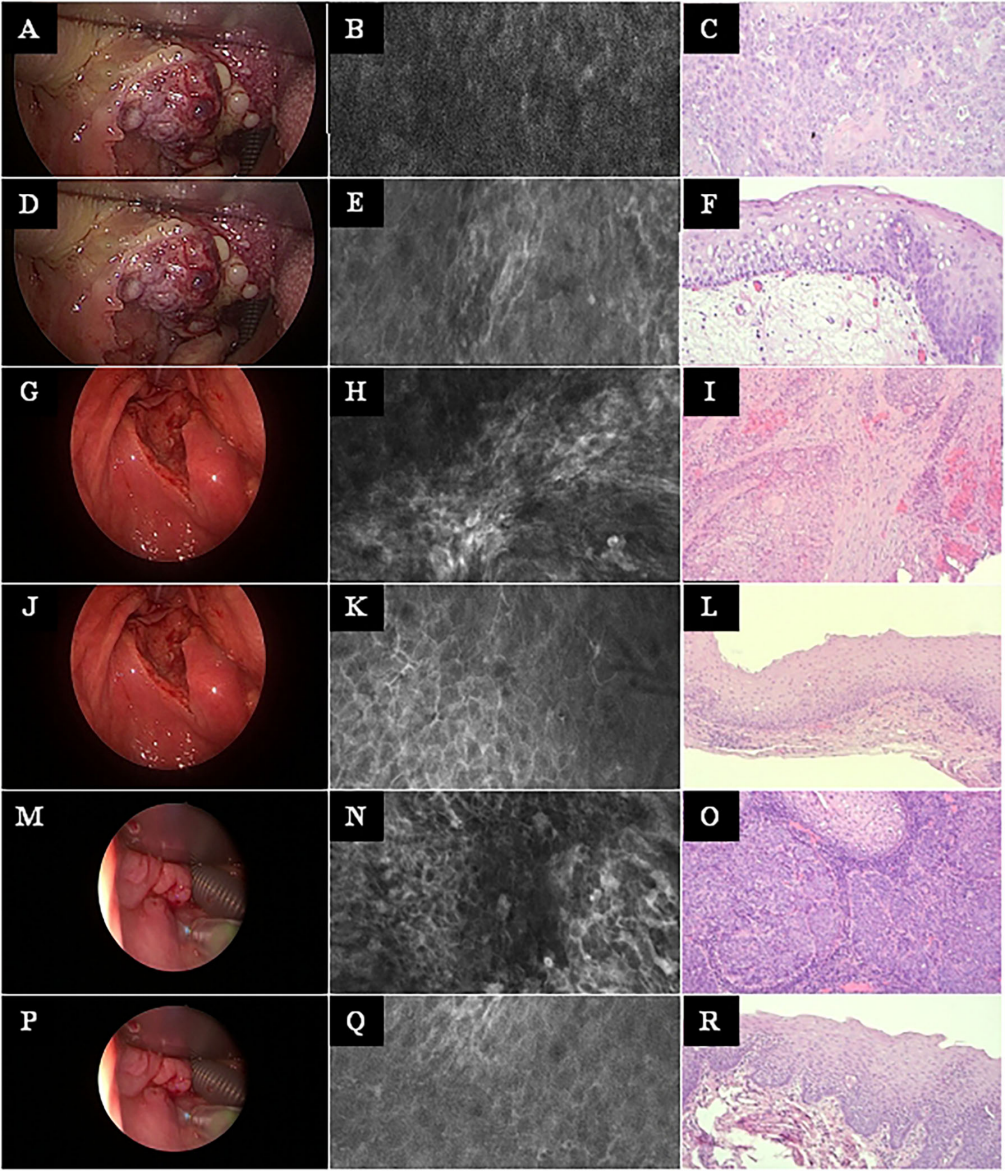
Head and neck squamous cell cancer of different subsites. Left row: White light endoscopy image. Middle row: Exemplary CLE image. Right row: Exemplary histopathology H&E image of the same tumor. **(A–C)** Tonsil cancer. **(D–F)** Same case as in A–C, now imaging and biopsy in the surrounding of the tonsil cancer. **(G–I)** Hypopharyngeal cancer. **(J–L)** Same case as in G–I, now imaging and biopsy in the surrounding of the hypopharyngeal cancer. **(M–O)** Another hypopharyngeal cancer. **(P–R)** Same case as in M–O, now imaging and biopsy in the surrounding of this hypopharyngeal cancer. CLE image size: 464 × 261 µm, 1,920 × 1,080 pixel. Histopathology: Hematoxylin & eosin staining, 10× magnification.

**Figure 6:**
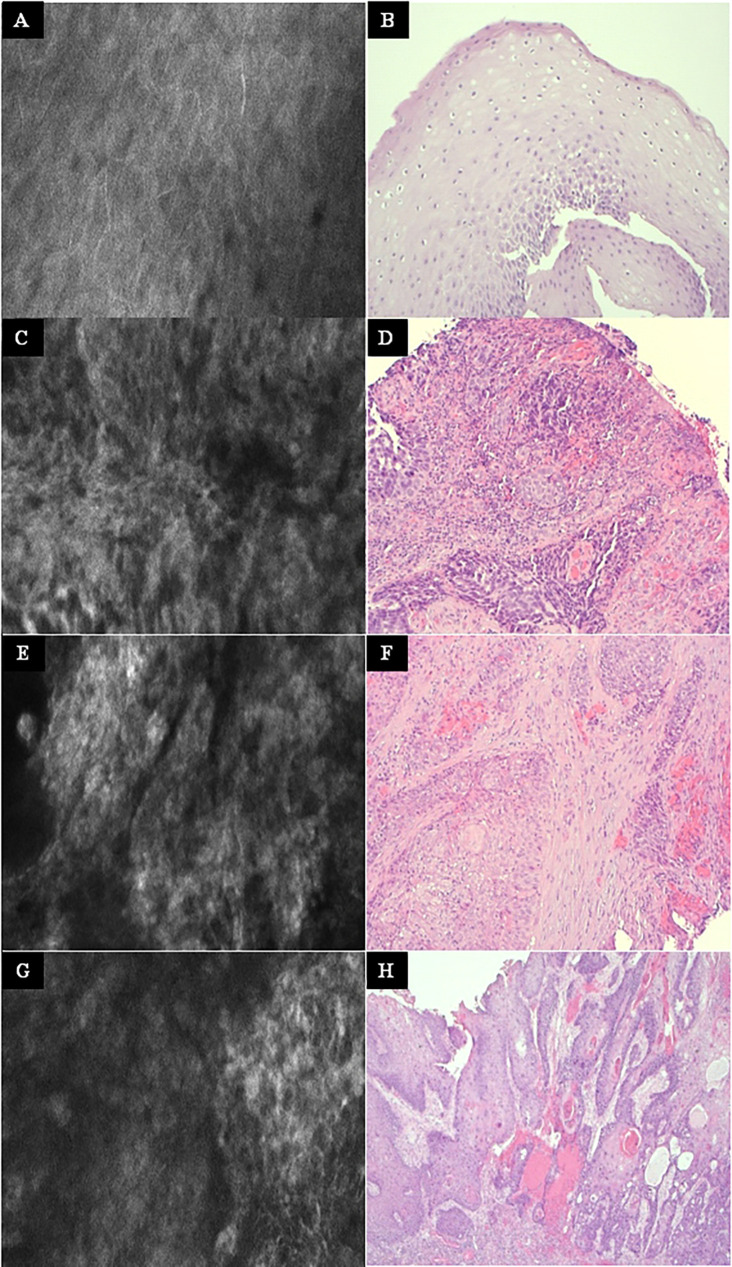
Other examples at higher magnification. Left column: Exemplary CLE image. Right column: Exemplary histopathology H&E image of the same tumor. **(A, B)** Normal mucosa in the oropharynx. **(C, D)** Tonsil cancer. **(E, F)** Hypopharyngeal cancer. **(G, H)** Oral cancer. CLE image size: 464 × 261 µm, 1,920 × 1,080 pixel. Histopathology: Hematoxylin & eosin staining, 10× magnification.

**Figure 7:**
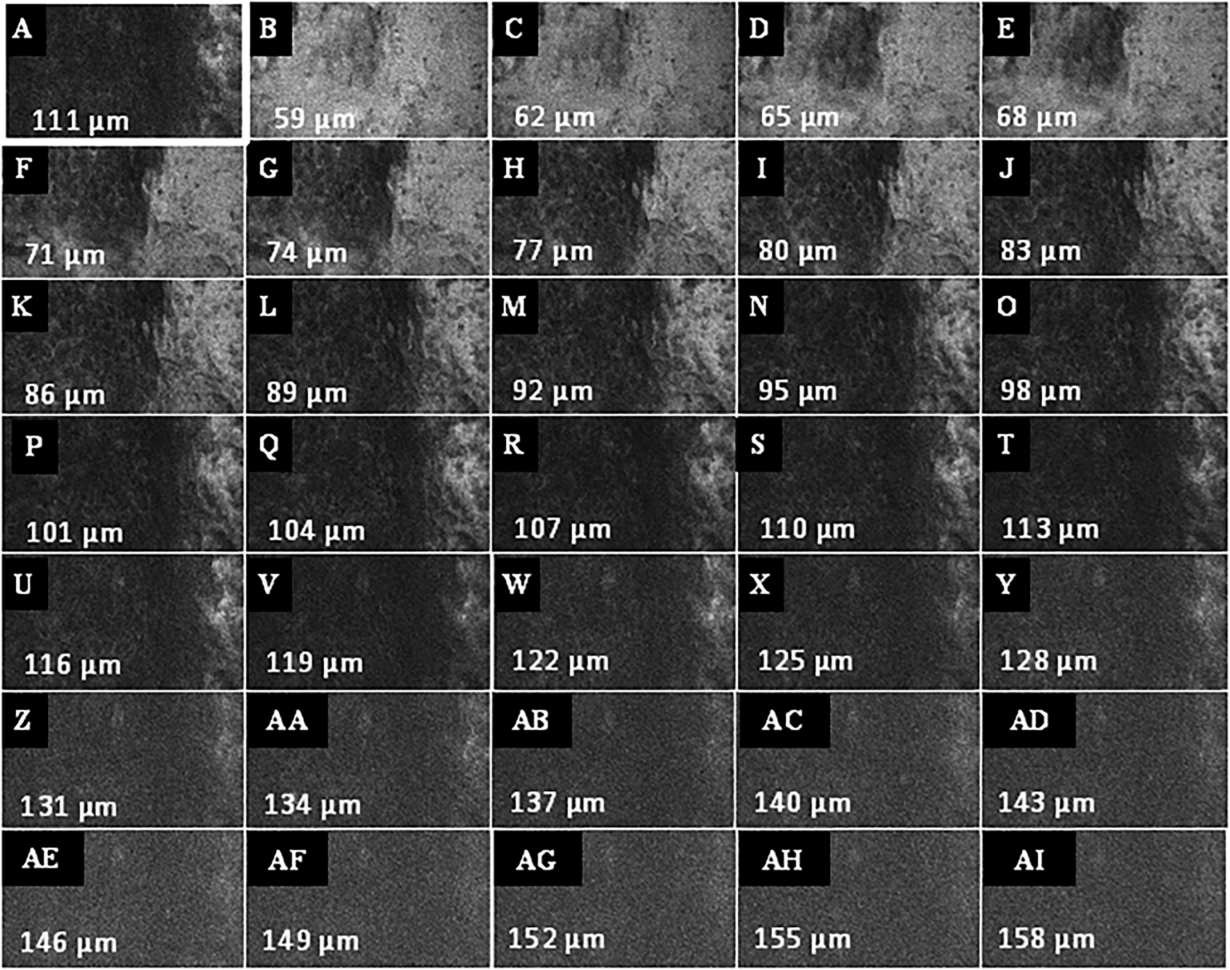
Example of a Z-stack acquisition in a patient with oropharyngeal carcinoma. **(A)** Definition of the first focus plane, here 111 µm below the mucosa surface of the tumor. **(B–AI)** Automated recording of the Z-stack in both directions (to surface and depth) relative to the current focal plane. The Z step, i.e. the interval of depth between the individual images, was set at 3 μm. CLE image size: 464 × 261 µm, 1,920 × 1,080 pixel.

**Figure 8:**
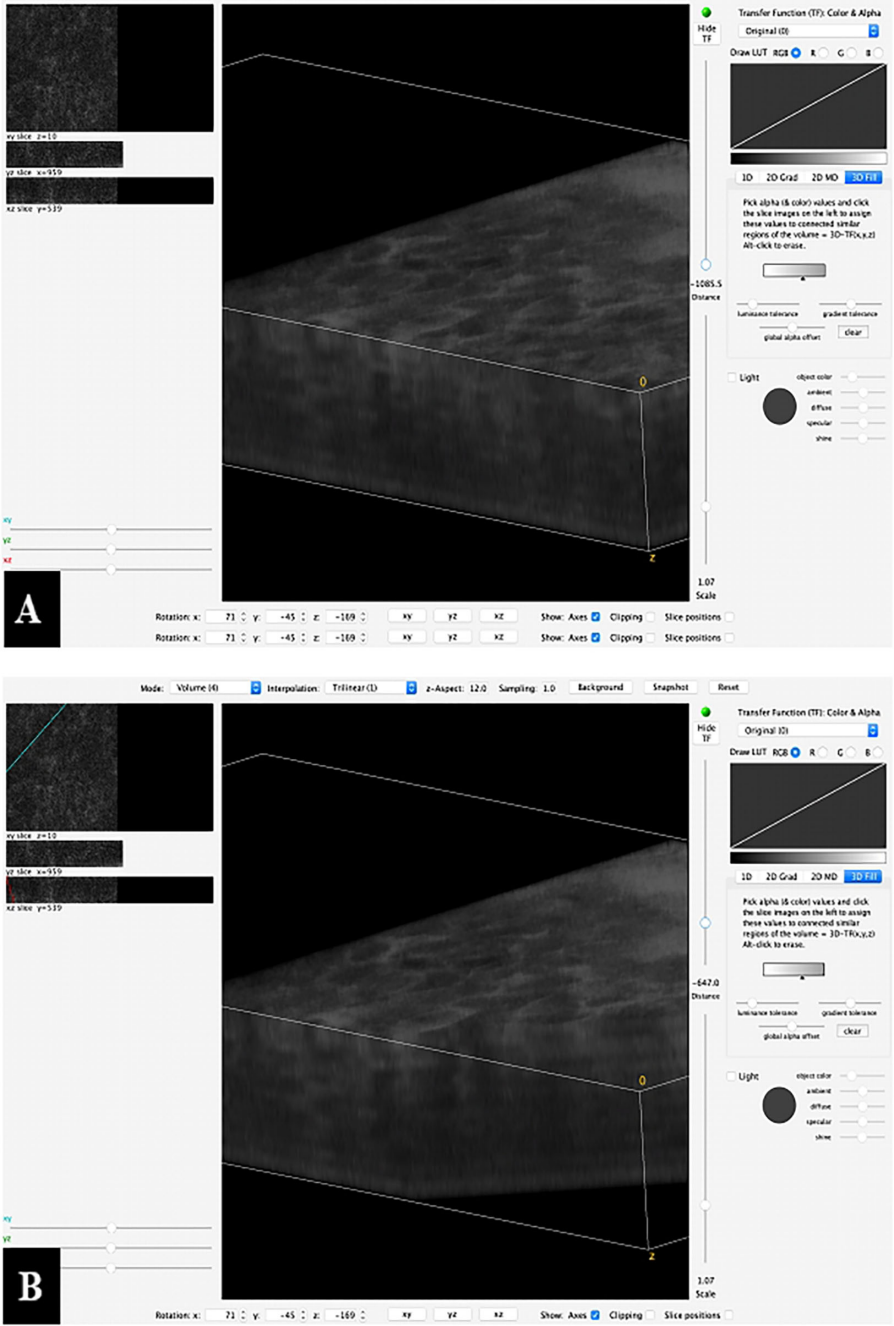
Example of a 3D reconstruction of the Z-stack from **Figure 6**. **(A)** All 35 images together in one stack with a horizontal view of the superficial mucosal layer and vertical slice view at a specific spot of the deeper layers. **(B)** The image processing software allows to scroll through the stack and shows the vertical slice view at each specific spot.

No complications or side effects caused by CLE or fluorescein use could be observed. The preparation of the intravenous fluorescein application and the CLE setup could be carried out parallel to the induction of anesthesia and therefore did not extend the operating time. Apart from the time of the video recording of the CLE itself (on average 9.0 ± 6.9 min), there was no relevant delay in the routine panendoscopy. A total of 2,565 CLE images were recorded. **Table 3** summarizes the details of the CLE image acquisition. Examples of CLE images of spots with histopathologically confirmed normal tissue, chronic inflammation, and cancer are showed in **Figure 3**. CLE images of normal tissue and chronic inflammation did not show obvious CLE differences. Three hundred twenty CLE images (12.5%) showed severe artefacts excluding a CLE tumor classification (**Figure 4**). A median of 178 images per patients were taken. A median of 68 CLE images per biopsy were taken. Examples for the patients with head and neck squamous cell carcinoma are shown in **Figures 5**, **6**. **Figure 7** shows an example of a Z-stack acquisition. Typically, using the minimal Z-step of 3 µm, interpretable CLE images were recorded between 65 and 120 µm from the mucosa surface. **Figure 8** shows a 3D reconstruction of the Z-stack from **Figure 7**. In-between the patients, the histopathological results annotated to each CLE image fitted to the CLE classification (malignant yes/no) due to the DOC-Score in 91.5 ± 5.9% of the images (cf. **Table 2**). The sensitivity, specificity, and accuracy to detect cancer in the biopsies using the classified CLE images was 87.5% (95% CI = 47.4–99.7), 80.0% (95% CI = 28.4–99.5), and 84.6% (95% CI = 54.6–98.1), respectively. The positive and negative predictive values were 87.5% (95% CI = 54.3–97.6) and 80.0% (95% CI = 37.8–96.3), respectively.

**Table 3:** Overview of the CLE image classification.

Parameter	Absolute (N)	Relative (%)
All	2,565	100
Artefact classification		
No artefacts	1,679	65.4
Minimal artefacts	566	22
Severe artefacts*	320	12.6
Annotated histology to images without/only minimal artefacts	2,245	100
Squamous cell carcinoma	1,324	58.9
Chronic inflammation	457	20.3
Dysplasia-free normal tissue	464	20.8
	**Mean ± SD**	**Median, range**
CLE images per patient	197 ± 181	178, 23–678
CLE images per biopsy	82.7 ± 47.1	68, 23–178

*Not allowing an evaluation of the image.

## Discussion

Using a median number of 178 CLE images per patients and 68 CLE images per biopsy, the *ex vivo* classification using the DOC-Score (12) blinded to the histopathological result allowed a correct assignment of a CLE image to show head and neck squamous cell cancer in contrast to normal mucosa in 91% of the images. Concerning the 13 patients of this pilot trial, this resulted in a sensitivity, specificity, and accuracy for tumor detection or exclusion of 87.5, 80.0, and 84.6%, respectively. This is in the range reported for other CLE systems: According to data of other studies, the sensitivity and specificity of diagnosing head and neck squamous cell cancer is reported to 85.0–95.3% and 72.0–100%, respectively (19, 20). Next step, a prospective trial with intraoperative *in-vivo* CLE classification compared to frozen section and final histopathology is needed. This study should also help to define the optimal number of biopsies and CLE images per biopsy spot. Another approach, less of interest for use during panendoscopy but during ablative tumor surgery, would be to apply CLE *ex vivo* on tissue probes taken for frozen sections (21).

Combing CLE imaging using a flexible probe-based CLE system with automated image analysis with deep learning for tumor detection, we previously reported for a series of 12 patients as specificity, sensitivity, and accuracy of 85, 72, and 74%, respectively (11). Recently, Aubreville et al. even reported an overall accuracy of 94.8% for automated CLE head and neck tumor detection. They also used a flexible CLE system and generated a data set of 15,000 images of the mucosa in the oral cavity and the vocal folds for a deep learning-based approach (22). Hence, another challenge will be to apply deep learning algorithms in the present setting to see if this outperforms an *in vivo* image interpretation. It will be of special interest to see, if the z-stack function allowing to add three-dimensional information and will therefore have additional value to improve the performance of the deep-learning approaches (23).

By use of the DOC-score (12), the focus of the present study was on the distinction between tumor and normal mucosa. The DOC-score was primarily developed to classify CLE images of the oral mucosa. The characteristics are not different in other head and neck areas (11). The CLE characteristics between normal mucosa and mucosa with chronic inflammation were not different. Moore et al. were even able to discriminate between normal non-dysplastic, dysplastic, and cancerous tissue (24). They defined a larger width variability of the epithelial lining as characteristic in CLE images of low-grade dysplasia. Moreover, collaboration with pathologists will help to extract more CLE characteristics out of the images to better define the multistep step from normal to cancerous tissue. It should be typical for high-grade dysplasia that the epithelial lining becomes irregularly thickened and more disorganized. We believe that these quantitative parameters have to be confirmed first by quantitative image analysis, and if confirmed, these parameters might be implemented in deep learning approaches. Furthermore, severe artifacts in the CLE images (motion, blood, saliva) are hindering or make it even impossible to classify the images (25). Deep learning approaches will also help to automatically deal with and sort out CLE images with severe artifacts (26–28).

Previous studies on head and neck cancer used CLE systems primarily designed for other disciplines (19). Fibered probe be integrated into an endoscope are mostly used (29). In contrast to the flexible probes, the CLE probe used in the present study consists of a handheld rigid probe with an outer diameter of 5 mm and a working length of 150 mm. The rigid probe appears to be more practicable and more suitable for scanning the mucous membrane in the oropharynx and hypopharynx. A disadvantage is the working length of the probe of only 150 mm, which makes the probe inaccessible for lesions in the larynx. Therefore, a longer rigid probe is needed. Another advantage of the rigid probe in comparison to the flexible systems on the market is penetration depth of 300 µm. If the z-stack function over such a large penetration depth is helpful for a better tumor border definition the depth up to the mucosal surface has to be investigated in future studies.

A relative new field is to use CLE also during open head and neck cancer surgery (30). We suppose that the handheld rigid probe design is also advantageous for an intraoperative CLE assessment of safe margins during open head and neck cancer surgery.

## Conclusion

CLE with a handheld rigid probe can be easily integrated into the intraoperative workflow of a panendoscopy. Beyond oral cancer, the applied CLE tumor classification score (DOC) was feasible also for other head and neck cancer subsites. The presented accurate *ex vivo* classification results have to be validated in further studies also *in vivo*. CLE seems to be a versatile technology enabling a more precise intraoperative tumor staging by better evaluation of the tumor margins.

## Data Availability Statement

The original contributions presented in the study are included in the article/**Supplementary Material**. Further inquiries can be directed to the corresponding author.

## Ethics Statement

The studies involving human participants were reviewed and approved by the ethics committee of the Jena University Hospital. The patients/participants provided their written informed consent to participate in this study.

## Author Contributions

AD, OGL: design of the work. AD, RZ, NG: data acquisition. All the authors: analysis and interpretation, draft contribution, and approval of the final version to be published; agreement to be accountable for all aspects of the work in ensuring that questions related to the accuracy or integrity of any part of the work are appropriately investigated and resolved. All authors contributed to the article and approved the submitted version.

## Conflict of Interest

The authors declare that the research was conducted in the absence of any commercial or financial relationships that could be construed as a potential conflict of interest.
