# Supplementary material for: Fluorescein-Guided Panendoscopy for Head and Neck Cancer Using Handheld Probe-Based Confocal Laser Endomicroscopy: A Pilot Study
Source: Front Oncol. 2021 Jun 14;11:671880. doi: 10.3389/fonc.2021.671880 (PMC8236705; doi:10.3389/fonc.2021.671880)
Supplement: Supplementary file 1 [file Table_1.docx]

**Supplement Table 1**

| Supplement Table 1. DOC-Score. Originally developed as CLE classification and scoring system of oral squamous cell carcinomas by Oetter et al.*. | | |
| --- | --- | --- |
| Evaluation criterion | Manifestation | Score |
| 1. Tissue architecture |  |  |
| 1.1. Homogeneity | Completely organized | 0 |
|  | Organized + unorganized regions | 1 |
|  | Completely unorganized | 2 |
| 1.2  Intercellular gaps | Regular | 0 |
|  | Mutated | 1 |
|  | No longer/non-existent | 2 |
| 2. Cell morphology | Consistent/regular | 0 |
|  | Inconsistent/dysplastic (different sizes/shapes/grey levels) | 1 |
|  | Completely irregular Dark, small cells Blurry, cloudy image Black spot (cell cluster) | 2 |
| 3. Fluorescence leakage | Regular | 0 |
|  | Amplified (bright background) | 1 |
| 4. Vessels | Regular | 0 |
|  | Irregular Shape (micro vessels, coiled, elongated etc.) Caliber (increased) Quantity (cumulated) | 1 |
| Maximal point |  | 8 |

x ≥ 5 points = squamous cell carcinoma (SCC)

*N. Oetter, C. Knipfer, M. Rohde, C. von Wilmowsky, A. Maier, K. Brunner, W. Adler, F. W. Neukam, H. Neumann and F. Stelzle: Development and validation of a classification and scoring system for the diagnosis of oral squamous cell carcinomas through confocal laser endomicroscopy. *J Transl Med*, 14(1), 159 (2016) doi:10.1186/s12967-016-0919-4
